# Epidemiological Survey of Human Alphaherpesvirus 2 (HSV-2) Infection in Indigenous People of Dourados Municipality, Central Brazil

**DOI:** 10.3390/tropicalmed8040197

**Published:** 2023-03-29

**Authors:** Flávia Freitas de Oliveira Bonfim, Livia Melo Villar, Julio Croda, Solange Rodrigues da Silva, Crhistinne Cavalheiro Maymone Gonçalves, Vivianne de Oliveira Landgraf de Castro, Grazielli Rocha de Rezende Romeira, Gabriela Alves Cesar, Sabrina Moreira dos Santos Weis-Torres, Marco Aurélio Horta, Simone Simionatto, Ana Rita Coimbra Motta-Castro, Vanessa Salete de Paula

**Affiliations:** 1 Virology Molecular Laboratory, Oswaldo Cruz Foundation, Rio de Janeiro 21040360, Brazil; 2 Viral Hepatitis Laboratory, Oswaldo Cruz Foundation, Rio de Janeiro 21040360, Brazil; 3 Brazilian Society of Tropical Medicine, Campo Grande 79070900, Brazil; 4 Binacional Campus of Oiapoque, Federal University of Amapá, Macapá 68903419, Brazil; 5 Center for Biological and Health Sciences, Blood Center Sector, Federal University of Mato Grosso do Sul, Campo Grande 79070900, Brazil; 6 State Department of Health of Mato Grosso do Sul, Campo Grande 79031350, Brazil; 7 Environmental Analysis Laboratory, Southern Cross University, Military Road East, Lismore, NSW 2480, Australia; 8 Biosafety Level 3 Platform (NB3), Oswaldo Cruz Foundation, Rio de Janeiro 21040360, Brazil; 9 College of Biological and Environmental Sciences, Federal University of Grande Dourados, Dourados 79070900, Brazil; 10 Center for Health and Biological Sciences, Oswaldo Cruz Foundation, Rio de Janeiro 79070900, Brazil

**Keywords:** human alphaherpesvirus 2, human herpes virus 2, indigenous, ethnicity

## Abstract

Sexually transmitted Human alphaherpesvirus 2 (HSV-2) causes genital ulcers, especially among sexually active adolescents and adults. We estimated the exact prevalence of anti-HSV-2 antibodies and correlated it with the demographic and behavioral aspects of the Indigenous population of the Jaguapirú and Bororó villages (Dourados, Mato Grosso do Sul (MS), Brazil). In total, 1360 individuals (>18 years old) were administered serologic tests. The prevalence of anti-HSV-2 IgM was 12.9%, that of anti-HSV-2 IgG was 57.2%, and 8.5% cases tested positive for both HSV-2 IgM and IgG. The prevalence of anti-HSV-2 antibodies was higher in females (59.5%) compared to males (49%), with an OR of 0.64 (0.49–0.83). Anti-HSV-2 antibodies were found in 14.2%, 12.3%, 15.4%, and 14.5% of participants with urinary problems, genital wounds, genital warts, and urethral discharge, respectively. In summary, the seroprevalence of HSV-2 in the Indigenous population was five times higher than that reported in the general adult Brazilian population. Educational level, income level, smoking, condom use, incarceration, illicit drug abuse, the sharing of used needles and syringes without adequate disinfection, homosexual relationships, prostitution, the sexual practices among drug users, and avoidance of contraceptive methods could contribute to the facilitation of HSV-2 transmission in the Indigenous population. Our results may help develop culturally appropriate intervention programs that eliminate health-access barriers and improve the implementation of public health policies aimed at promoting information regarding and preventing, treating, and controlling HSV-2 infection in Brazilian Indigenous populations.

## 1. Introduction

Although the Indigenous population of the Dourados municipality in the Mato Grosso do Sul (MS) state of Brazil is predisposed to a high risk of acquiring Human alphaherpesvirus 2 (Human herpes virus 2 or HSV-2), HSV-2 prevalence data are lacking for this ‘priority-population’. According to the 2010 census (conducted by the Brazilian Institute of Geography and Statistics (IBGE) and the National Indian Foundation (FUNAI)), Brazil has an Indigenous population of approximately 896,917 individuals, with 324,834 living in urban areas and 572,083 living in rural areas; Currently about 1.3 million Indigenous people in Brazil [1]. Overall, the present Indigenous population encompasses 305 ethnicities and 274 languages, spread over 12.5% of the Brazilian territory [1]. The Indigenous population studied in Mato Grosso do sul (MS), which is located in the central region of Brazil [1], is the second-largest indigenous population in Brazil [2]. The ethnic groups present in MS are the Atikum, Guarani-Kaiowá, Terena, KiniKinawa, Kadiwéu, Guató, Guarani-Nhandeva, and Ofaié [3]. Inhabiting the Jaguapirú and Bororó villages of Dourados city, the largest ethnicities are represented by the Guarani-Kaiowá and Terena ethnicities, comprising about 18,000 people in an area of 3474.50 ha [4,5].

The risk behaviors and vulnerability factors that lead to increases in the prevalence of infection diseases, specifically HSV-2, among the Indigenous population of the Dourados/MS area include the proximity of Indigenous populations to urban centers and international borders, interactions with non-Indigenous people in society, the urban presence of Indigenous youth, alcoholism, illicit drug use, resistance to condom use, unprotected sex, the lack of access to health information and diagnostic services, poverty, rituals involving the sharing of inadequately disinfected sharp objects, polygamy, cross-breastfeeding [6,7,8,9], and changes in sexual behavior patterns [10,11]. The scarcity of epidemiological data specific to Indigenous communities greatly contributes to the higher HSV-2, morbidity and mortality rates in these areas compared to those in the general population in Brazil [11,12]

Herpesviridae family viruses infect several species and are widely distributed worldwide. HSV-2 transmission occurs through bodily fluids such as blood and saliva, primarily in sexual intercourse, with an increase during puberty [13]. These viruses are also responsible for several diseases in humans; further, asymptomatic/undiagnosed cases persist as reservoirs facilitating transmission [14,15,16,17,18].

Human Alphaherpesvirus 2 or Human herpesvirus 2 (HSV-2) is the main cause of genital herpes, one of the most prevalent STIs worldwide (both in developed and developing countries). HSV-2 is the most prevalent infection in the world, and in Brazil, there are 640,000 new cases of genital herpes diagnosed annually [19]. Relevant public health data and the analysis of antibody prevalence allows for the identification of the dynamics of this epidemic [20]. HSV-2 establishes a latent infection in lumbosacral sensory and autonomic ganglia or sacral ganglia innervating the genitals and persists throughout life. Recurrent genital HSV-2 lesions can also arise from latent virus reactivated in sensory cell bodies of the dorsal root ganglia (DRG). The spontaneous reactivation of chronic infection can affect peripheral nervous tissues, and the presence of this infectious virus in these tissues helps viral transmission [21]. Reactivation by various stimuli (such as immunosuppression, stress, or hormonal changes) can trigger clinical symptoms [22,23,24]. As a result, asymptomatic or widespread episodes with recurrent painful ulcerations (which increase the risk of acquiring human immunodeficiency virus (HIV)) of the genital mucosa may occur [14,25,26,27]. Both primary and recurrent HSV 2 infections in pregnant women can lead to intrauterine transmission and can result in congenital HSV infection [28]. HSV-2 is correlated with a serious risk of vertical infection or mother-to-child transmission during childbirth [29] and is excreted in breast milk in a significant proportion of postpartum women; accordingly, breastfeeding may be an important route for the transmission of HSV-2 to babies [6]

Herpes simplex virus type 2 (HSV-2) affects approximately 22% of adults 12 years and older, representing 45 million adults in the United States. While HSV-1 commonly affects the perioral region and can cause genital lesions, HSV-2 more commonly presents in patients with genital lesions. Accordingly, a large portion of patients affected by HSV infection outbreaks present with nonspecific symptoms, such as genital itching, irritation, and excoriation, which can delay diagnosis and treatment. As a consequence, exposure to uninfected healthy individuals may occur [30].

The seroprevalence of HSV-2 antibodies in the general population in Brazil was 11.3%, accounting for a total of 1090 individuals aged 1 to 40 years [20]. In 2017, HSV-2 prevalence in America was 29.6%. Interestingly, higher age (within the infection-bracket) is directly associated with greater exposure to HSV-2, although primary infection most often occurs in the adolescent phase, starting at age 15. Newborns infected with HSV-2 in the birth canal may present with rashes on the skin, mouth, and eyes, or present more severe conditions such as splenomegaly, hepatomegaly, kidney failure, jaundice, neurological damage, and encephalitis [18,31,32,33,34].

We aimed to evaluate the prevalence and behavioral aspects of HSV-2 in the Indigenous population of the Dourados/MS Reserve using serological samples from Jaguapirú and Bororó villages. Our survey is useful for the development of culturally appropriate programs that may contribute to facilitating access to public health services, eliminating stigmata concerning the transmission and treatment of herpes, and supporting the implementation of public health policies regarding the promotion, prevention, treatment, intervention, and control of HSV-2 infection in the Brazilian indigenous population.

## 2. Materials and Methods

### 2.1. Ethical Statement

All subjects gave their informed consent for inclusion before they participated in the study. The study was conducted in accordance with the Declaration of Helsinki, and the study protocol was approved in March 2017 by the Ethics Committee of the University of Grande Dourados (UFGD-MS) (CAAE:62012616.3.0000.5160 (number 2.000.496)).

This was a retrospective, cross-sectional, analytical study. This study was conducted in two villages in the municipality of Dourados, Mato Grosso do Sul (MS), Brazil. Between March 2017 and November 2018, the Indigenous population data and blood samples were collected by a team of trained health professionals, such as doctors, physicians, biologists, and nurses, who were aided by an interpreter fluent in the native language so as to clarify the procedure and help address any doubts.

Only Indigenous individuals aged >18 years with mental and intellectual abilities sufficient for understanding the study were included. All participants signed a free and informed consent form and answered the socio-epidemiological questionnaire individually, thereby guaranteeing the privacy and anonymity of each participant. The multidisciplinary Dourados Indigenous Area Health Team (EMSI, Bororó, and Jaguapirú 1 and 2) in charge of providing primary health care services supported this study in various fashions, including aiding translation.

Our sampling team (composed of doctors and nurses) underwent two training sessions (with the indigenous health agents and teams) before the study began, and blood extraction was performed through venipuncture using sterile equipment and needle. In addition to pre-training, we had the structured questionnaire validated through interviews with Indigenous health professionals. When necessary, the indigenous health agent translated the questionnaire into the native language when the indigenous person could not speak Portuguese. Subsequently, this questionnaire was used in the study. The interview conducted using the questionnaire aimed to profile the participants in terms of their risk and the protective factors inherent in the Indigenous population to understand the current reality of the study context. The questionnaire covered—among other variables listed as significant—sociodemographic information; history of drug and alcohol use; medical history, presence of signs and symptoms related to hepatitis B and C, HIV, and herpes infections; housing conditions; and income and education status. Individuals >18 years of age residing in the study area and who had signed an informed consent form were included in the study. Individuals providing blood sample volumes that were insufficient for the performance of the anti-HSV-2 test were excluded from the study. Our study required these variables, which are fundamental to the relationship between HSV-2, its prevalence, and socio-demographic relationships.

### 2.2. Serological Analysis

Serological markers of HSV-2 infection in blood samples were detected using anti-HSV-2 (gG2) IgM and anti-HSV-2 (gG2) IgG enzyme-linked immunosorbent assay (ELISA) at Euroimmun (Euroimmun Diagnóstico Médico—Laboratorial Brasil). The sensitivity and specificity of the immunoassays was 100% (according to the manufacturer’s instructions and manufacturer’s own positive, calibrator, and negative control). The results were evaluated using categorized outcomes (positive and negative for HSV-2 IgG and IgM, respectively). As per the protocol, the quality of the antigen used ensures the high specificity of the ELISA and that cross-reaction with anti-HSV-1, which latter frequently reacts with anti-HSV-2 and thus causes false positives, does not occur. Anti-HSV-2 (gG2) ELISA (IgM) order no EI 2532-9601-1 M LOTE: E180525CE and Anti-HSV-2 (gG2) ELISA (IgG) order no EI 2532-9601-2 G LOTE: E190716AZ.

### 2.3. Statistical Analysis

Data from the Special Indigenous Health District of Mato Grosso do Sul (SIHD/MS) were used to determine the total study population. According to data provided by the SIHD/MS, 13,094 Indigenous people live in Bororó and Jaguapirú villages in the municipality of Dourados/MS, of which 6291 are >18 years of age. Considering a 20% loss related to refusals, the population size eligible for sampling was estimated to be 3400, including men and women. The Indigenous population of these two villages comprised the Guarani-Kaiowá, Terena, Kadiwéu, and Guarani-Nhandeva ethnic groups.

To calculate the sample size adjusted for finite populations, we used the following formula: $n=\left(Z^{2}*\;P\;\left(1-P\right)\right)/E^{2},$ where *Z* is the value of the standard normal distribution corresponding to the desired confidence level (Z:1.96 and IC 95% CI); *P* is the expected prevalence; and *E* denotes desired precision (half of the desired IC). Considering a 20% loss related to refusals, the population size eligible for sampling was estimated to be 295, including men and women, generating a total that was 4.6 times (1360 study subjects) greater than the estimated sample population.

The socio-epidemiological information on the indigenous population investigated in this research was collected; thus, the data and information were added to the existing database for statistical analysis and sent to the Ministry of Health of Mato Grosso do Sul to initiate awareness and treatment campaigns of individuals reactive for HSV-2. The data were divided into several categories/blocks: Block A—general information; Block B—sociodemographic information; Block C—history of drug and alcohol use; Block D—tuberculosis; Block E—STI status; and Block F—Test performed. Responses to the questionnaires were in the form of Yes or No answers. The questionnaire was validated by the ethics committee of the University of Grande Dourados (UFGD-MS) and included as Supplemental Information.

Data concerning age, gender, and socio-epidemiological information were tested against serological status using the Pearson chi-square test. Prevalence was obtained, and the prevalence odds ratio (POR) or odds ratio (OR) were estimated to assess the association of sociodemographic variables with HSV-2 positivity among Indigenous people. The data were stored in Excel spreadsheets and exported to RStudio (version 2022.02.3) for statistical analysis. Maps of the study site, case distribution, and spatial analysis were created using QGIS (version 3.26.1), employing computers provided by (property number F-IOC-54641) the the Oswaldo Cruz Foundation, Rio de Janeiro, Brazil.

## 3. Results

In the current study, the sample population included 1360 (>18 years old) Indigenous individuals, among whom 77.6% were females (1056/1360) with an age range from 18 to 103 years, while the male proportion accounted for 22.4% (304/1360) with an age ranged from 18 to 83 years.

**Figure 1:**
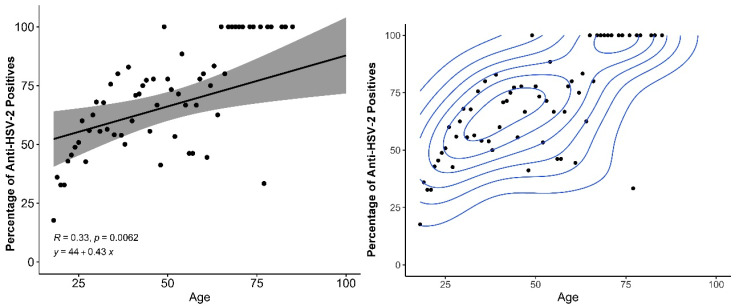
Relationship between age of participants and percentage of HSV-2 positive individuals, with linear increase (**left**) and density estimation with two separated age groups (**right**).

A high prevalence of HSV-2 IgG was observed (57.2%); females showed significantly greater prevalence of HSV-2 IgG (59.5%) compared to males (49%), as shown in Table 1. Figure 1 plots the distribution of HSV-2 positivity vs. age, where in the mean age of infection was 40.6 years with a concentration of HSV-2 prevalence around 29 and 39 years.

**Figure 2:**
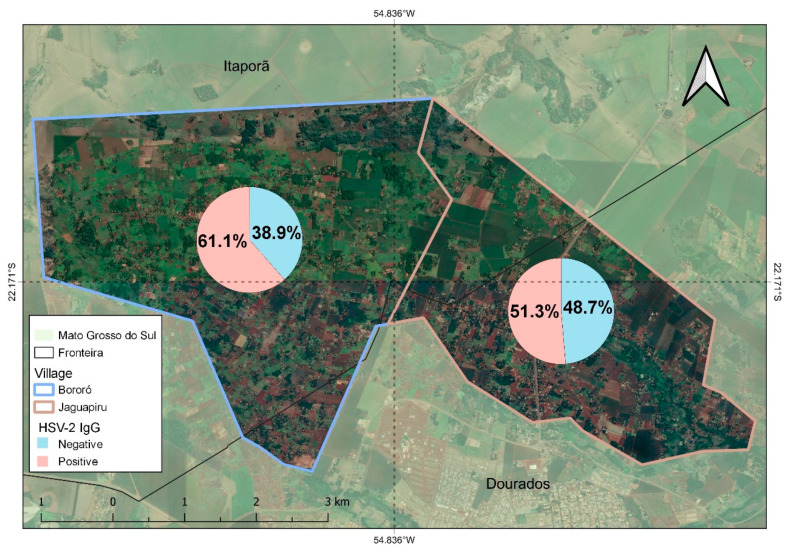
Distribution of anti-HSV-2 IgG prevalence positive and negative prevalence in Bororó and Jaguapirú villages (Dourados, Mato Grosso do Sul (MS), Brazil).

Bororó villagers (61.1% (495/805)) had significantly higher HSV-2 IgG prevalence than Jaguapirú villagers (51.3%, O.R 0.67 (0.53–0.88)), as shown in Table 1, Figure 2. We found that socio-demographic variables affected the anti-HSV-2 IgG prevalence. The prevalence of anti-HSV-2 IgG in Indigenous people declared retired was significantly higher ((73.2%) OR of 2.1 (1.36–3.45) (*p*-value 0.001)) compared to the Indigenous population who were non-retired, the prevalence was 55.8% HSV-2 IgG.

The use of technological devices such as cell phones and HSV-2 IgG prevalence was reported by 55.1% with an O.R of 0.73 (0.57–0.94) and a *p*-value of 0.01, and internet use was observed in 30.9% with an O.R of 0.41 (0.4–0.54) and a *p*-value less than 0.001; this can be compared to the proportions of Indigenous people who did not use the internet (69%) and cell phones (44.8%).

**Figure 3:**
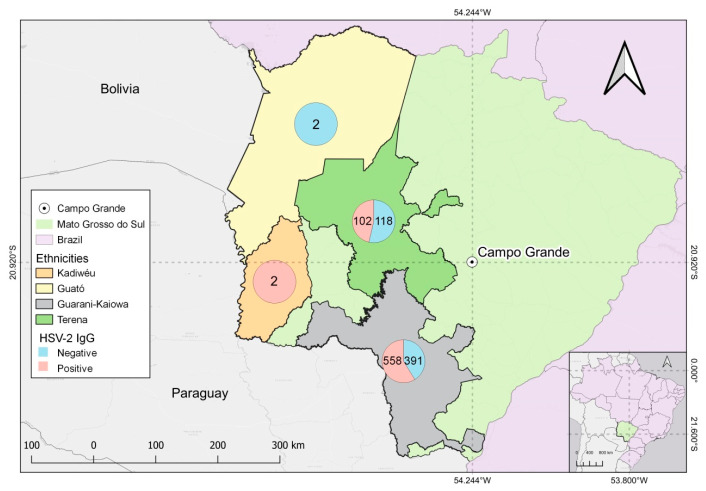
Positive and negative outcomes for anti-HSV-2 IgG serological test among Indigenous Guarani-Kaiowá, Kadiwéu, Guató, and Terena ethnicities and their geographical locations in Mato Grosso do Sul, Brazil.

The difference between anti-HSV-2 IgG prevalence in the Guarani-Kaiowá (58.8%) and Terena (46.3%) was statistically significant. The difference in anti-HSV-2 IgG prevalence in other ethnicities, such as the Guarani-Nhandeva and Kadiwéu, whose prevalence values were 50% and 100%, respectively, was not statistically significant (Figure 3). Education status greatly affected anti-HSV-2 IgG prevalence. Elementary-educated (61.2%), and high-school-educated (43.6%) individuals had significantly higher prevalence than the college-educated (34.5%) population (Table 2). Families earning >5 minimum wages had a lower prevalence of anti-HSV-2 IgG (27.3%) than families earning 1–2 minimum wages (53.5%) (Table 1). Anti-HSV-2 IgG prevalence among condom users (sexually active) was 52.9%, which was significantly higher than non-condom users (47%) with an O.R 0.75 (0.60–0.93).

However, anti-HSV-2 IgG prevalence in former prisoners (60.5%), alcoholics (54.3%), illicit drug users (44.4%), tattoo-bearers (56.0%), syringe- and needle-sharers (57.4%), those indulging in sexual intercourse with a non-injecting illicit drug user (79.6%), those indulging in sexual intercourse with an injecting drug user (41.7%), sex workers (64.7), and those engaging in homosexual relationships (61.5%) was not statistically different compared to the other risk factors mentioned above.

In Table 2, the Terena ethnic group showed the highest rates of individuals in Elementary School (14.1%) and High School (22.6%), which were different from the Guarani-Nhandeva and Guarani-Kaiowá ethnic groups. However, the Guarani-Kaiowá ethnic group presented the highest rate of indigenous individuals with a college education (50.6%) and the highest rates of income of the other ethnic groups earning one to two minimum wages (64.6%), three to four minimum wages (49.3%), and one to two minimum wages (54.5%). The Guarani-Nhandeva ethnic group registered the lowest levels of income and education.

Among the clinically symptomatic Indigenous individuals, anti-HSV-2 IgM was detected in 14.2%, 12.3%, 15.4%, and 14.5% of those exhibiting urinary problems, genital wounds, genital warts, and urethral discharge, respectively (Table 3). Anti-HSV-2 IgM was found in 14.4% (115/805) and 10.8% (60/555) of Indigenous individuals from Bororó and Jaguapirú villages, respectively. Totals of 13.8% (130/949), 9.1% (20/220), and 7.8% (5/18) of Guarani-Kaiowá, Terena, and Guarani-Nhandeva, individuals exhibited clinical symptoms and reacted for anti-HSV-2 IgM.

## 4. Discussion

STI prevalence is related to the vulnerability and risk behaviors of the studied indigenous population. Epidemiologically, cultural and social risk behaviors such as sexual history, sexual partners, familial incomes and education, ethnicity, gender, age, condom use, events involving the sharing of contaminated sharp objects, sexual initiation, alcoholism, socioeconomic condition, and past history of an STI can contribute to the high prevalence of HSV-2. Vulnerability of populations to HSV-2 is increased by proximity to national and international borders, the geographical area, and other factors [35].

Among the sample population, anti-HSV-2 IgM prevalence was 12.9% (176/1360) and about 14% declared having some type of history of genital sores, including urethral discharge (14.5%), urinary problems (14.2%), genital wounds (12.3%), and genital warts (15.4%), which were among the most frequent signs and symptoms of HSV-2 infection, where in most could be asymptomatic. In a study conducted of São Paulo, Brazil, only 4.3% of pregnant women and 21.6% of patients with STIs that were seropositive for HSV-2 had a clinical history of genital herpes [36]. According to Amudha et al., approximately 20% of herpes infections have vesicular or ulcerative lesions in the genital region. In addition, more than half of the infection form is asymptomatic, facilitating transmission to healthy individuals [37]. HSV-2 is the main cause (55.3%) of ulcerations in the Indigenous and non-Indigenous Brazilian patients from the Amazon region [38]. We found that 8.5% cases tested positive for HSV-2 IgM and IgG, indicating that genital herpes symptoms may be caused by initial or recurrent infection [37]. Epidemiological data for HSV-2 in populations with a history of genital herpes revealed that the disease is underestimated because of asymptomatic cases, thereby facilitating its transmission to healthy individuals [36]. The prevalence of HSV-2 IgM reveals that the HSV-2 outbreak may have occurred in the Bororó and Jaguapirú indigenous populations, with a high rate of reactivation or primary infection by HSV-2 IgM as there was an unexpected increase in infection in this specific region [38]

Here in, a high prevalence of HSV-2 IgG was identified in Brazilian Indigenous populations. The HSV-2 IgG prevalence was significantly higher in women (59.5%) than in men (49%), with an OR of 0.64 (0.49–0.83). Thus, the sexual transmission of HSV-2 is more efficient from men to women than from women to men [39,40]. Moreover, women typically have higher seroprevalence rates for HSV-2 than men [41,42,43]. The high prevalence rates of HSV-2 infections in women of childbearing age may present as a risk of inducing neonatal herpes [43].

Figure 1 shows that with increasing age, the percentage of positive tests increases according to a statistically significant relationship among those over 18 years of age. Furthermore, it shows that two sets of ages are very well separated, in the older age groups, there are a higher percentage of positives with increasing age. Older individuals (in the age-bracket) have an increased risk and prevalence of HSV-2, which progressively increases with age. Time in years of sexual activity and sex with multiple partners are determinants of an increased risk of HSV-2 infection [44]. Seroprevalence increased with age—in adolescence and among young adults—with a sensitivity of 2.2% in HSV-2 seropositive individuals in the general population with a clinical history of genital herpes and 14.3% in individuals with a history of STIs. Epidemiologic studies of HSV2 that base their conclusions on a history of genital herpes largely underestimate the problem [20]. HSV-2 prevalence increases with age, with older people in villages generally having a higher chance of acquiring the virus due to their longer history of sexual activity, thus representing a higher risk Moreover, HSV-2 IgG prevalence in retired Indigenous individuals was significantly higher was 73.2% (OR 2.13 (1.36–3.45)) compared to those who were not retired by 27%. The requirements for an indigenous person to be retired are age (men over 60 or women over 55 who had at least 15 years of proven work), disability or accident retirees, had a high and The requirements for an indigenous person to be retired are age (men over 60 or women over 55 who had at least 15 years of proven work), disability or accident retirees, had a high prevalence, and the prevalence increases with increasing age [45].

The presence of untreated, disseminated HSV-2 infections can lead to high mortality rates in high-risk groups (such as Indigenous populations) with inherently higher risk and vulnerability to STIs (mainly because of their cultural practices). Due to their characteristic latency period, herpesviruses can persist indefinitely in the host, transmitting to other healthy natives after activation either asymptomatically or symptomatically [46,47,48].

In 2016, an estimated 491.5 million individuals in the general population worldwide (95% uncertainty interval, UI: 430.4 million–610.6 million) were living with HSV type 2 infection, equivalent to 13.2% of the world’s population aged 15–49 years. Different trends by age, sex, and geographic region were observed in the study by James et al., wherein HSV 2 prevalence was highest among women in the WHO African Region [49].

In this study, IgG was prevalent in 57.3% of HSV-2 infections, which is 1.5× higher than the Brazilian national prevalence. In his research on the cities of Rio de Janeiro, Manaus, Porto Alegre, and Ceará in Brazil, Clemens evidenced that seroprevalence of HSV-2 antibodies in the general population in Brazil (11.3%) amounted to a total of 1090 individuals (aged 1–40 years) [20]. Thus, from this study, the HSV-2 prevalence in Indigenous populations in Dourados/MS reserve is 5x higher than that in the non-Indigenous Brazilian population. In a 2015 Brazilian study, HSV-2 prevalence was as high as 30% in non-Indigenous adults depending on the age at first sexual activity [20,40]. Further, HSV-2 IgG prevalence (59.7%) in this study is similar to that seen in HIV/HSV-2 pregnant women. However, the HSV-2 IgM level in the Indigenous population (12.9%) is higher than that seen in HIV/HSV-2 pregnant women (6%) [47,48].

The HSV-2 prevalence rates in the present study (57.3%) are about 5 and 4.3 times (13.2%) higher than those observed in the general population of the world and Brazil (11.3%), respectively; a gradual increase in prevalence rates was also observed with increasing age (within the affected age-bracket) of the Indigenous population. The Indigenous population in Australia had significantly higher prevalence of HSV-2 (18%) than the general non-Indigenous (12%). Prevalence in Australia of HSV-2 was highest in the 35–44 year age range (14–19%) in comparison with the youngest age group (25–34 years) [50]. Early onsets of sexual activity and culturally specific practices seen in Australians have contributed to the widespread increase in HSV-2 prevalence and other STIs. HSV-2 prevalence observed in the Indigenous population was higher than that in non-Indigenous sex workers in the central western region of Australia (47.3%) [51].

Among the villages studied, Bororó had significantly higher HSV-2 prevalence than the Jaguapirú, at 61.1% and 51.3%, respectively (OR 0.67 (0.53–0.88). This indigenous reserve lies in Dourados city (Mato Grosso do Sul), comprising the Bororó and Jaguapiru villages, which are mostly represented by the Guarani-Kaiowá and Terena ethnicities [3,4]. Adherence to the study was higher in the Bororó population possibly due to the difficulty in accessing the comparatively distant health services. Traditionally, Bororó village extends from Bolivia to the Miranda River in Brazil [52]. The morbidity rate of the Bororó population reflects the precariousness of its living conditions; the main causes of morbidity are infections linked to basic sanitation, hygienic habits, and alcoholism. The Indigenous populations of Jaguapirú and Bororó comprise families from dozens of Indigenous communities, such as Guanari-Kaiowá, Guarani-Nhandeva, Terena, Guató, Kadiwéu, and other ethnic groups, also including Paraguayans and regional Brazilians who are assimilated by interethnic marriages [52].

We found that anti-HSV-2 IgG prevalence among the Guarani-Kaiowá was 58.8% and 46.3% for the Terena, which was statistically significant with ORs of 1.32 (1.04–1.6) and 0.60 (0.45–0.81). The anti-HSV-2 IgG prevalence in the ethnicities Guarani-Nhandeva and Kadiwéu was 50% (9/18) and 100% (2/2), respectively, and was not statistically significant. The Guarani-Kaiowá ethnicity had significantly higher (58.8% OR 1.32 (1.04–1.6) HSV-2 prevalence, indicating that belonging to this ethnic group increases the risk of acquiring HSV-2 compared to other ethnic groups in the study. The Terena ethnicity had significantly lower HSV-2 prevalence (46.3%, OR 0.60 (0.45–0.81)) compared to other ethnic communities, indicating that belonging to this community protects one from anti-HSV-2 infections. In Table 2, the Terena ethnic group has higher rates of individuals in Elementary School (14.1%) and High School (22.6%), However, the Guarani-Kaiowá ethnic group has the highest rate of indigenous people with a college education (50.6%) and presented higher income rates in relation to the other two ethnic groups at 1 to 2 minimum wages (64.6%), 3 to 4 minimum wages (49.3%), and 1 to 2 minimum wages (54.5%). The Guarani-Nhandeva ethnic group presented the lowest levels of income and education. The Terena and Guarani-Kaiowá ethnic groups are the most represented in the two study villages, which are located about 5 km from the city of Dourados, which provides access to the urban center, free of contact with non-indigenous populations. [2,52].

The Indigenous people travelling/migrating between villages had anti-HSV-2 IgG prevalence of 59.6% (OR 1.41 (1.06–1.87)) and a 41% higher risk of having anti-HSV-2 IgG than those who did not travel/migrate. HSV-2 prevalence was significantly affected by education status. The HSV-2 prevalence was 34.5% (0.33 (0.18–0.57), 43.6% (OR 0.48 (0.56–0.63), and 61.2% among college-educated, high-school-educated, and elementary-educated populations, respectively. Thus, the higher the level of education, the lower the prevalence of HSV-2, which is a protective factor against infection, thus revealing the connection between low education and HSV-2 seroprevalence [42,53,54,55].

The higher the salary of the Indigenous people, the lower the HSV-2 prevalence and, consequently, the lower the chances of acquiring the virus (OR 0.32 (0.07–1.14). The prevalence of several diseases is related to conditions of poverty and low wages, as well as scarce access to information and health services, which are currently accessible via the internet on electronic devices [53,54,55,56]. Furthermore, the HSV-2 prevalence of 55.5% (OR 0.73 (0.57–0.94)), 52.2% (OR 0.69 (0.55–0.86)), and 30.9% (OR 0.41 (0.4–0.54)) in Indigenous people who used cell phones, television, and the internet, respectively, was significantly higher when compared to that in the respective non-device-users. Thus, individuals with access to communication media were more likely to acquire HSV-2. This finding reinforces the notion that a lack of information and access to diagnostic services may favor the acquisition of HSV-2. It is important to emphasize that the difficult access and remoteness of some villages limits their access to media. Communication and information are essential for disease awareness and prevention [56].

Smokers had higher HSV-2 prevalence, namely, 61%, compared to that in non-smokers, which was 48.6% (OR 1.34 (1.08–1.6). Smoking appears to be an independent factor and one not associated with poverty and alcohol use, which are also important risk factors for contracting HIV, STIs and Tuberculosis [57,58,59]. The HSV-2 prevalence among Indigenous individuals who self-reported condom use (52.9%) (sexually active) was higher than in those who did not use condoms (47% OR 0.75 (0.60–0.93)). The use of condoms is a protective factor, as condom use is directly linked to the prevention of HSV-2 and other STIs [60,61,62,63,64,65]. The risk of unprotected sex (occasionally or never using condoms) among sexual partners of the same ethnicity, concurrent partners, and partners using illegal drugs is associated with low frequency of consistent condom use and, in turn, vulnerability to the transmission of STIs other than HSV-2 in indigenous migrant agricultural workers [64,66]. Behaviors that generate risk directly facilitated the acquisition of HSV-2; the HSV-2 prevalence was high with respect to the use of illicit drugs, alcoholism, the use of sharp objects, and having a tattoo, amounting to 44.4%, 54.3%, 57.4%, and 56%, respectively. Low condom use can also be considered a possible contributor to increased STI rates, with only 37% reporting condom use. Behaviors such as indulging in sex work, engaging in sexual encounters outside cultural contexts, and repeated break-ups are additional risk factors. The behavioral characteristics of the Indigenous culture analyzed in this study facilitate a high risk of acquiring and transmitting HSV-2 [62,63,67,68]. Having unprotected sex along with alcoholism and/or drug use increases the risk of sexually transmitted infections such as HSV-2 [69].

Through the research presented herein, it is evident that the control and prevention of HSV-2 must be made a public health priority in the villages of Jaguapirua and Bororó in Mato Grosso do Sul, Brazil. The corresponding strategies should be drawn based on population-specific epidemiological information; distinctions in infection profiles in the general and ‘at-risk’ populations (such as the Indigenous people studied herein) must be considered. Finally, our results emphasize (a) the importance of epidemiological surveillance with respect to strategizing prevention and control measures for HSV-2, and (b) the need for public policies accounting for the realities of the Indigenous Brazilian population and their specific-risk behaviors for HSV-2 acquisition.

## 5. Conclusions

The seroprevalence of HSV-2 in Indigenous populations was five times higher than that reported for the general adult Brazilian population. We identified high prevalence of HSV-2 infection in Jaguapirú and Bororó village populations. The prevalence of HSV-2 IgM reveals the rate of reactivation or primary infection for HSV-2 IgM, which possibly caused the outbreak of HSV-2 that occurred in the Bororó and Jaguapirú indigenous populations. The presence of untreated, disseminated HSV-2 infections can lead to high mortality rates in high-risk groups. Educational level, income level, smoking, condom use, former incarceration, illicit drug abuse, the sharing of used needles and syringes without adequate disinfection, engaging in homosexual relationships, prostitution, sexual practices with drug users, and the avoidance of contraceptive methods could contribute to the widespread increase in HSV-2 infection. Furthermore, In addition, the use of condoms in sexual relations among indigenous peoples needs to be a goal for the awareness of these priority populations in a way that is respectful in culture. Our results will help (1) develop culturally appropriate intervention programs that can contribute to eliminating health-access barriers and (2) implement public health policies aimed at promoting information on HSV-2 infections and preventing, therapeutically intervening, and controlling the virus among the Indigenous population of Brazil.

## Figures and Tables

**Table 1:** Comparison of different variables associated with anti-HSV-2 IgG prevalence among Indigenous communities in Bororó and Jaguapirú villages (Dourados, Mato Grosso do Sul (MS), Brazil).

Variable	N	N Positive	Prevalence (%)	*p*-Value *	POR ** (IC 95%)
Village				<0.001	
Jaguapiru	555	285	51.3%		0.67 (0.53–0.88)
Bororó	805	495	61.1%		
Gender				<0.001	
Female	1056	629	59.5%		
Male	304	149	49.0%		0.64 (0.49–0.83)
Lived in another village previously	255	152	59.6%	0.02	1.41 (1.06–1.87)
Family allowance	667	403	60.4%	0.01	1.3 (1.05–1.61)
Retired	97	71	73.2%	0.001	2.13 (1.36–3.45)
Cell phone	999	551	55.1%	0.01	0.73 (0.57–0.94)
Television	644	336	52.2%	0.001	0.69 (0.55–0.86)
Internet	281	87	30.9%	<0.001	0.41 (0.4–0.54)
Ethnicities					
Guarani-Kaiowá	949	558	58.8%	0.02	1.32 (1.04–1.6)
Terena	220	102	46.3%	0.001	0.60 (0.45–0.81)
Guarani-Nhandeva	18	9	50.0%	0.69	
Kadiwéu	2	2	100%	0.61	
Guató	2	-	-	0.35	
Education				<0.001	
Elementary School	859	526	61.2%		
High School	296	129	43.6%		0.48 (0.56–0.63)
College education	55	19	34.5%		0.33 (0.18–0.57)
Working	413	217	52.5%	0.02	0.76 (0.6–0.96)
Smoking	656	398	60.7%	0.008	1.34 (1.08–1.6)
Use of Condoms	504	266	52.9%	0.01	0.75 (0.60–0.93)
Income				0.02	
1 to 2 minimum wages	494	263	53.5%		
3 to 4 minimum wages	71	28	39.4%		0.56 (0.33–0.93)
>5 minimum wages	11	3	27.3%		0.32 (0.07–1.14)
Former inmate	38	23	60.5%	0.81	
Alcoholism	348	189	54.3%	0.21	
Illicit drogs	45	20	44.4%	0.10	
Tatoo	339	188	56.0%	0.60	
Syringe and needle sharing	163	93	57.4%	0.99	
Sexual intercourse with a partner who is a non-injecting illicit drug user	110	65	79.6%	0.68	
Sexual intercourse with partner who is an injecting drug user	12	5	41.7%	0.42	
Sex worker	17	11	64.7%	0.70	
Homosexual relationship	26	16	61.5%	0.81	

* *p*-values from the chi-squared test. ** Crude Prevalence Odds Ratio.

**Table 2:** Relationship between HSV-2 prevalence, ethnicity, socioeconomic, and education variables in Indigenous populations of Bororó and Jaguapirú villages (Dourados, Mato Grosso do Sul (MS), Brazil).

Variable	Guarani-Kaiowá		Guarani-Nhandeva		Terena	
Income	N (%)	*p*-value *	N (%)	*p*-value	N (%)	*p*-value
		0.03		0.22		0.22
1 to 2 minimum wages	319 (64.6)		9 (1.8)		108 (21.9)	
3 to 4 minimum wages	35 (49.3)		2 (2.8)		22 (31.0)	
>5 minimum wages	6 (54.5)		1 (9.1)		3 (27.3)	
Education		<0.01		0.11		<0.01
Elementary school	639 (0.74)		8 (0.9)		121 (14.1)	
High school	172 (0.58)		6 (2.0)		67 (22.6)	
College education	28 (50.9)		2 (3.6)		17 (30.9)	

* *p*-value estimated using Pearson’s chi-squared test.

**Table 3:** Seroprevalences of HSV-2 (anti-HSV-2 IgM) in individuals exhibiting clinical symptoms in Indigenous community, Mato Grosso do Sul, Brazil.

	N	N Positive	Prevalence	*p*-Value *
Urinary problems	155	22	14.2	0.7
Genital Wounds	57	7	12.3	-
Genital Warts	26	4	15.4	0.93
Urethral discharge	138	20	14.5	0.65

* *p*-values from the chi-squared test.

## Data Availability

The data presented in this study are available upon request from the corresponding author. All data from this research have been reported in this article.

## Supplementary Materials

The following supporting information can be downloaded at: https://www.mdpi.com/article/10.3390/tropicalmed8040197/s1, Questionnaire: Indigenous Population.
