# Supplementary material for: Epidemiological Survey of Human Alphaherpesvirus 2 (HSV-2) Infection in Indigenous People of Dourados Municipality, Central Brazil
Source: Trop Med Infect Dis. 2023 Mar 29;8(4):197. doi: 10.3390/tropicalmed8040197 (PMC10143357; doi:10.3390/tropicalmed8040197)
Supplement: Supplementary file 1 [file tropicalmed-08-00197-s001.zip › tropicalmed-2027384-supplementary.pdf]

FEDERAL UNIVERSITY OF GRANDE DOURADOS
GRADUATE PROGRAM IN HEALTH SCIENCES
MULTICENTRIC STUDY OF INFECTIOUS DISEASES IN THE INDIGENOUS POPULATION OF THE DOURADOS-MS RESERVE

BLOCK A - GENERAL INFORMATION

1. Questionnaire number: __ __ __ __

2. Responsible for data collection :___________________________

3. Date of data collection : ____/____/_____

4. Typist : _____________________

5. Typing date : ____/____/____
6. City: _________________

7. Village: ______________________________

8. Residence/House : ____________________________

HOW IS YOUR HOME? ( ) Wood ( ) shack ( ) brick

9. Type of housing?____

How do you get water?

10. Does the house have running water?
(1) Yes ______
(2) No _____
11. If the answer is no, how is the water supply?____
_

12. Name: ____

11. How long have you lived in the village?
(months)____

12Which village did you previously live in?____

13. What religion? ____

14. Do you receive benefits such as family allowance and retirement?
(1) Yes ______
(2) No _____
15. What benefits do you receive?____

16. What means of communication do you use?
( ) Cell Phone
( ) Radio
( ) TV
( ) internet

17. Gender: __

Ask for identification document, to be reliable data

18. Birth date : ___/___/___

BLOCK B - SOCIAL DEMOGRAPHIC INFORMATION

19. Naturalness : ________________________

20. What state were you born in?__ __

21. What is your ethnicity? __ **(1) Guarani-Kaiwá (2)Guarani-Nhandeva (3) Terena (4) Kadiwéu (5) Guató (6) Kinikinaw (7) Ofaié (8) Other (______________)**

22. You're? __ (1) Married or living with a partner (2) Widowed (3) Separated/divorced (4) Single

Until what year did you study?

23. What was the last school grade you attended? ? ______________________

23. It works? (1) Yes (2) No

24. What is the monthly income of everyone in the family: R$ __________ per month

25. How many people live with you? __ __

26. Have you ever been arrested? __ (1) Yes (2) No

BLOCK C – NUTRITIONAL HISTORY

29. What's your weight? __ __ __

30. What's your height? __ __ __

31. Do you take any medication? __ (1) Yes (2) No

32. If yes, specify which medication you use? _______________

Drug and alcohol history

33You smoke? __ (1) Yes (2) No. If not, skip to question 37**.**

34. If yes, at what age did you start? __ __

35. If yes, how many cigarettes do you smoke per day? __ __

If the respondent smokes, skip to question 37.

36. Have you ever smoked? __ (1) Yes (2) No

Have you ever used any of the following drugs:

	Have you used it in the last year?<br>(1) Yes<br>(2) No
Alcohol	37.
Marihuana	38.
Cocaine	39.
Crack (stone)	40.
Heroin	41.
Sniffed glue/other solvents	42.
Base paste	43.
Hashish	44.
Did you inject any drugs? Which are:	45.

If male: If not, skip to question 53.

46. Have you ever had a prostate exam? ( ) Yes No

47. If so, which one? ( ) digital rectal examination ( ) blood test (psa) ( ) ultrasound ( ) biopsy

48. Do you have cancer in the family? ( ) Yes No

49. If so, what cancer was diagnosed?____

50. Is your diet high in fat? (   ) Yes No

51. Do you have any urinary problems such as burning, weak stream, waking up at night to urinate or blood in the urine? (   ) Yes No

52. If so, which ones?____

BLOCK D - TUBERCULOSIS

History of tuberculosis-related signs and symptoms

53. Do you have or have you had tuberculosis before? __ (1) Yes (2) No (3) Don't know. If not go to question 100
54. How many treatments were performed?

55. How long ago was the last treatment performed?__ __ months.

56. How long ago was the last treatment performed?__ __ months. Type of discharge (last): __ (1) Cure (2) Abandonment (3) Don't know

57. Do you know someone with TB? __ (1) Yes (2) No (3) Don't know.

BLOCK E – SEXUALLY TRANSMITTED INFECTIONS

58. Do you have or have you had a sexually transmitted disease? __ (1) Yes (2) No (3) Don't know
If not go to question 70

59. How long ago was the last treatment performed?____ months.

60. Do you have HIV?__ (1) Yes (2) No

61. If so, are you on antiretroviral treatment?

62. Do you have hepatitis? __ (1) Yes (2) No

63. Do you have syphilis? __ (1) Yes (2) No

64. If yes, are you being medicated? __ (1) Yes (2) No

65. Have you ever had syphilis?__ (1) Yes (2) No

66. If so, did you take all three doses of the drug? __ (1) Yes (2) No

67. If so, did the partner take all three doses of the drug? __ (1) Yes (2) No

68. Has a test been offered for these diseases (HIV, viral hepatitis, syphilis? __ (1) Yes (2) No

69. If yes, for which:____________________

70. Have you ever had a blood transfusion? __ (1) Yes (2) No

71. If so, what year?_________

72. You have tattoo? __ (1) Yes (2) No

73. If yes, how many?

74. Do you have a piercing? __ (1) Yes (2) No

75. Do you have a piercing? __ (1) Yes (2) No

76. Do you have a sore on your penis or vagina? __ (1) Yes (2) No

77. Do you have or have you had urethral discharge? __ (1) Yes (2) No

78. Do you have or have you had warts on your penis or vagina? __ (1) Yes (2) No

79. Have you ever had sexual intercourse with a non-injecting illicit drug user partner? __ (1) Yes (2) No

80. Have you ever had sex with an injecting drug user? (1) Yes (2) No

81. Have you ever had sex with a partner with HIV? __ (1) Yes (2) No

82. Have you ever had sexual intercourse with a partner with syphilis? __ (1) Yes (2) No

83BR Have you ever had sexual intercourse with a partner with hepatitis? __ (1) Yes (2) No

84. Do you have a steady sexual partner? __ (1) Yes (2) No

85. If yes, for how many years?

86. What is the number of partners in the last 5 years? __ __

87. Are you a sex worker? __ (1) Yes (2) No

88. Have you ever been a sex worker? __ (1) Yes (2) No

89. Have you ever had sex for money or drugs? __ (1) Yes (2) No

90. What is your sexual preference? __ (1) homosexual (2) heterosexual

91. If you are heterosexual, have you ever had a homosexual relationship? __ (1) Yes (2) No

92. Do you use condoms during sexual intercourse? __ (1) Yes (2) No

93. Have you ever shared syringes/needles? __ (1) Yes (2) No

94. Have you ever shared objects for tattooing, pliers, shaving equipment, for using inhaled drugs? __ (1) Yes (2) No

95. Have you ever had any surgery? __ (1) Yes (2) No

96. If so, what year?_______

97. Have you ever had a hepatitis B vaccine? __ (1) Yes (2) No

98. If yes, how many doses?

BLOCK F - EXAMINATIONS CARRIED OUT - post-testing

<u>Serologies</u>

99. Blood collection date: __/__/____

100. Rapid test for syphilis: __ (1)Reagent (2) Non-reactive

101. Rapid HIV test: __ (1)Reactive (2) Non-reactive

102. Hepatitis B rapid test: __ (1)Reactive (2) Non-reactive

103. Hepatitis C rapid test: __ (1)Reactive (2) Non-reactive

104. Anti-HIV 1/ 2: __ (1) Reagent (2) Non-reactive

105. INNO-LIA III HCV Ab: __ (1)Reagent (2) Non-reactive

106. Western Blot for HIV: __ (1)Reactive (2) Non-reactive

107. Anti-Treponema pallidum IgG: __ (1)Reagent (2) Non-reactive

108. Anti-*Treponema pallidum* IgM: __ **(1)Reagente (2) Não-reagente**<br><br>109. HBsAg: __ **(1)Reagente (2) Não-reagente**<br><br>110. Anti-HBs: __ **(1)Reagente (2) Não-reagente**<br><br>111. Anti-HBc total: __ **(1)Reagente (2) Não-reagente**<br><br>112. Anti-HCV: __ **(1)Reagente (2) Não-reagente**	
